# Improving access to Chagas disease diagnosis and etiologic treatment in remote rural communities of the Argentine Chaco through strengthened primary health care and broad social participation

**DOI:** 10.1371/journal.pntd.0005336

**Published:** 2017-02-13

**Authors:** Paula Sartor, Ivana Colaianni, M. Victoria Cardinal, Jacqueline Bua, Héctor Freilij, Ricardo E. Gürtler

**Affiliations:** 1 Universidad de Buenos Aires, Consejo Nacional de Investigaciones Científicas y Técnicas, Instituto de Ecología, Genética y Evolución de Buenos Aires (IEGEBA), Facultad de Ciencias Exactas y Naturales, Buenos Aires, Argentina; 2 Dirección de Epidemiología, Ministerio de Salud de la Provincia del Chaco, Chaco, Argentina; 3 Programa Nacional de Chagas, Ministerio de Salud de la Nación, Buenos Aires, Argentina; 4 Hospital Santojanni, Buenos Aires, Argentina; 5 Instituto Nacional de Parasitología "Dr. M. Fatala Chaben", ANLIS C.G. Malbrán, Buenos Aires, Argentina; US Food and Drug Administration, UNITED STATES

## Abstract

**Background:**

Rural populations in the Gran Chaco region have large prevalence rates of *Trypanosoma cruzi* infection and very limited access to diagnosis and treatment. We implemented an innovative strategy to bridge these gaps in 13 rural villages of Pampa del Indio held under sustained vector surveillance and control.

**Methodology:**

The non-randomized treatment program included participatory workshops, capacity strengthening of local health personnel, serodiagnosis, qualitative and quantitative PCRs, a 60-day treatment course with benznidazole and follow-up. Parents and healthcare agents were instructed on drug administration and early detection and notification of adverse drug-related reactions (ADR). Healthcare agents monitored medication adherence and ADRs at village level.

**Principal findings:**

The seroprevalence of *T*. *cruzi* infection was 24.1% among 395 residents up to 18 years of age examined. Serodiagnostic (70%) and treatment coverage (82%) largely exceeded local historical levels. Sixty-six (85%) of 78 eligible patients completed treatment with 97% medication adherence. ADRs occurred in 32% of patients, but most were mild and manageable. Four patients showing severe or moderate ADRs required treatment withdrawal. *T*. *cruzi* DNA was detected by qPCR in 47 (76%) patients before treatment, and persistently occurred in only one patient over 20–180 days posttreatment.

**Conclusions and significance:**

Our results demonstrate that diagnosis and treatment of *T*. *cruzi* infection in remote, impoverished rural areas can be effectively addressed through strengthened primary healthcare attention and broad social participation with adequate external support. This strategy secured high treatment coverage and adherence; effectively managed ADRs, and provided early evidence of positive therapeutic responses.

## Author summary

Less than 1% of patients infected with *Trypanosoma cruzi* have access to parasiticidal treatment with the two available drugs, including millions of patients in the early chronic phase who would greatly benefit from treatment. Furthermore, rural populations living under poor and marginalized conditions usually have little or no access to chemotherapeutic programs of Chagas and other neglected tropical diseases. Barriers to treatment range from misconceptions on medication-related risks to socio-cultural aspects, among others. This study addressed the challenge of diagnosis and treatment of *T*. *cruzi* infection in resource-poor, remote rural settings of the Argentine Chaco where 24% of residents up to 18 years of age were infected. The underlying premise was that participatory methods and multisector cooperation would increase program effectiveness. Our results show that diagnosis and treatment of *T*. *cruzi* infection there can be effectively addressed through strengthened primary healthcare attention and broad social participation with adequate external support, following an initial phase of intensified vector control and surveillance across the municipality. This strategy secured high diagnosis-and-treatment coverage and adherence; effectively managed adverse drug-related reactions; provided early evidence of a positive therapeutic response, and may stimulate healthcare services in the affected regions to aggressively bridge the treatment gap.

## Introduction

Chagas disease ranks among the main neglected tropical diseases (NTDs) in Latin America and the Caribbean [1]. *Trypanosoma cruzi*, its etiologic agent, induces heart and digestive disease and reduces life expectancy in approximately 30–40% of the infected people [2,3]. The parasite infects 6–9 million people, the majority of which primarily were rural residents living in poverty with little access to healthcare services [4]. A well-known hotspot of Chagas disease and other NTDs is the Gran Chaco ecoregion which mainly extends over sections of Argentina, Bolivia, and Paraguay [5]. In rural villages across this region where *Triatoma infestans* is the only domestic vector, the seroprevalence of human *T*. *cruzi* infection in children younger than 15 years of age frequently ranged between 20% and 50% [6–12].

Prevention of human *T*. *cruzi* infection has traditionally relied on residual insecticide spraying campaigns and routine screening of blood-bank donors [2]. The two drugs (nifurtimox and benznidazole) registered for treatment of human infection with *T*. *cruzi* since the late 1960s and early 1970s were shown to be especially effective in young age groups during the acute and early chronic phase regardless of transmission mode [3,13–18]. Unfortunately, both nifurtimox and benznidazole cause adverse drug-related reactions (ADR) of various types, frequency and severity which increase with increasing patient’s age and reduce treatment compliance and effectiveness [19–22]. Benznidazole frequently causes mild or moderate dermatitis that respond well to antihistamines; low-dose oral glucocorticoids are less frequently needed. The rare cases presenting severe exfoliating dermatitis, dermatitis combined with fever and lymphadenopathy, and bone marrow suppression prompt immediate treatment discontinuation and intensive medical care [16,19,22,23]. Therefore, chemotherapeutic programs of human *T*. *cruzi* infection ideally should provide access to diagnosis and treatment as early as possible during the life course, minimize the occurrence of ADRs leading to reduced medication adherence, and avert eventual life threats posed by severe ADRs in the absence of timely medical attention.

Less than 1% of patients infected with *T*. *cruzi* have access to parasiticidal treatment [24]. Most populations living under poor and marginalized conditions often lack access to diagnosis and treatment of *T*. *cruzi* infection (and other neglected diseases) and are unaware of their condition [25]. They also ignore disease consequences and the opportunities and limitations of current therapies. Barriers to treatment are multiple and include lack of training on ADR management, misconceptions on medication-related risks, reluctance to provide treatment, wide fluctuations in medication availability, overburdened or distant healthcare services, socio-cultural aspects, and lack of effective vector control and surveillance [22,26–29].

The pioneering treatment programs of *T*. *cruzi* infection implemented by Médecines Sans Frontières (MSF) in various countries since 1999 demonstrated that the challenge was tractable with adequate resources and stringent procedures [21]. They proposed that “Etiological treatment of Chagas disease can and should be integrated at the primary health care level…” [21]. This recommendation has also been endorsed by others [3] and may be traced back to the Declaration of Alma Ata in 1978 [30]. The proposition is also related to the concept of innovative and intensified disease management (IDM) for NTDs that can be managed within the primary healthcare system through more intensive use of existing tools, as is the case of Chagas disease [31]. However, the challenge of how to address diagnosis and treatment of *T*. *cruzi* infection in resource-poor, remote rural settings through primary health care has yet to be developed and program effectiveness documented to meet the challenge of treating the sizable population of infected rural residents and correct health inequities.

The primary healthcare model focuses on community participation and social empowerment [30]. Broad social participation of multiple sectors may augment the feasibility and sustainability of control interventions, more so in disperse rural areas including various cultural groups [32–34]. Community participation is expected to increase the coverage, effectiveness and sustainability of vector and disease control actions of Chagas and malaria [35–39]. For example, in a remote rural area of Santiago del Estero (Argentine Chaco) under sustained community-based vector control in the mid-1990s, 17 (65%) of 26 *T*. *cruzi*-seropositive children aged up to 15 years of age treated with benznidazole or nifurtimox seroconverted to a negative status between 2 and 13 years posttreatment [39].

As part of a long-term program on the eco-epidemiology and control of Chagas disease in the Argentine Chaco, we developed, implemented and tested a strategy to increase access to diagnosis and treatment of human *T*. *cruzi* infection in sparsely populated rural sections of Pampa del Indio municipality including 13 villages. This strategy, based on strengthened primary healthcare attention and broad social participation, followed an initial phase of intensified vector control and surveillance across the municipality [40–42]. The underlying premise was that participatory methods and multisector cooperation combined with adequate external support would increase diagnosis-and-treatment coverage and adherence relative to historical local levels, manage ADRs effectively and achieve positive therapeutic responses, as we document in this paper.

## Materials and methods

### Ethics statement

The study protocol was approved and supervised by “Dr. Carlos Barclay Independent Ethical Committee for Clinical Research”, Buenos Aires, Argentina (Protocol N° TW-01-004). All clinical investigations were conducted according to the principles expressed in the Declaration of Helsinki. The Ministry of Health of Chaco province and local hospital authorities granted permission to conduct the activities herein described. All individuals participating in serosurveys and treatment accepted to do so and their parents or guardians provided written informed consent. When community meetings included indigenous residents, explanations were translated by an indigenous healthcare agent or by an appointed indigenous community member, and consent was obtained collectively and individually.

### Study area and transmission status

The intervention was conducted in Pampa del Indio (25°55’S 56°58’W), Chaco, Argentina. The study area included 353 houses and a few public buildings grouped in 13 rural villages distributed over a 450 km^2^ section as described elsewhere [40]. The study area was inhabited by 1,187 people in 2007 (1,318 people as of 2012, including 565 up to 18 years of age), most of which lived on a subsistence economy. The only existing medical facility was a first-level public hospital with four physicians; the primary healthcare system had 5 posts distributed across the study area, and there were 8 primary schools. The closest and farthest villages were at 6 and 37 km from the hospital through dirt roads, and there was no public transportation.

Initial selection of the study area followed the recommendations of the Chagas disease control program of Chaco: lack of insecticide spraying campaigns over the previous 12 years; reportedly high infestation levels with *T*. *infestans*, and requests of vector control interventions made by local district and healthcare authorities. Following an initial survey which provided evidence of large house infestation and active parasite transmission, all inhabited house compounds were sprayed with pyrethroid insecticides in November-December 2007 [40,43]. House infestation with *T*. *infestans* was monitored every 4–6 months from 2007 to 2010 (which revealed moderate pyrethroid resistance levels) and annually thereafter; all houses found to be reinfested were selectively re-sprayed with insecticides after each survey [40]. House infestation at the time of human diagnosis and treatment (September 2010-March 2011) was <1% and mainly occurred in peridomestic structures; none of the bugs collected were infected with *T*. *cruzi* as determined by microscopic analysis of feces at 400×. These conditions and an established vector surveillance system were taken as prerequisites to launch diagnostic and treatment activities.

### Program design

The diagnosis-and-treatment program included five successive phases: preparatory, participatory planning, capacity strengthening of local health personnel, diagnostic surveys, and treatment and follow-up.

#### Preparatory phase

Preliminary meetings with local authorities, health personnel, rural school teachers, and community leaders were conducted to inform them of the planned intervention program and request their feedback.

#### Participatory planning

Community workshops were conducted to introduce the research team, communicate the program’s objectives and phases, identify local capacities and weaknesses linked to healthcare activities, and establish a permanent channel of communication with local referents (S1 Table). Householders of each target village were convened by radio broadcasts and written messages to schoolchildren’s parents at each rural school. Participants included local residents, school teachers, healthcare agents, and leaders of social organizations. The workshops were conducted at six local healthcare posts or schools used as meeting points; each lasted an average of 1.5 h. A consensus was built on the following topics: blood samples would be drawn at the rural schools given their widespread presence; dates for serosurveys and delivery of results were established; further communications were through radio messages or cell phones if urgent (the study area had network coverage, and at least one cell phone was available at each village), and treatment with benznidazole and follow-up were to be conducted at the residents’ villages and included the active participation of rural healthcare agents.

#### Capacity strengthening of health personnel

Three meetings with local health personnel (including physicians, technicians, nurses and healthcare agents) were conducted to canvas their experience on attending Chagas disease patients and to identify locally available resources, including diagnostics and logistics (S2 Table). Printed leaflets with project information were distributed to consolidate the main messages. Meeting outcomes were used to define more precisely the roles of rural healthcare agents and coordination team members, and to adapt field activities to the detected constraints (S2 Table). Major obstacles identified were related to transportation and access to the study communities since the hospital had only one ambulance with an overburdened schedule; the solution was to dedicate one of the research team’s vehicles to diagnosis and treatment activities. The second obstacle identified was how to warrant close monitoring of patients during the drug administration period and provide an adequate response to any relevant ADR; the solution was to provide specific training to healthcare agents, include the new task in their work schedule, and instruct the patients’ parents on the type of ADR that may occur and how to respond.

#### Diagnostic surveys

Residents from all the study villages were summoned to the nearest healthcare post or school for blood sample extraction between November 2010 and January 2011. Blood samples were drawn by venipuncture from all residents older than one year of age (3 mL from children aged 1–2 years old, and 5–7 mL from older patients) who signed the informed consent. Capillary blood was taken from infants aged 9–12 months by fingerprick or heal prick. The decision to perform venipuncture (rather than collecting whole blood on filter paper, Serokit (Polychaco) or using a rapid test) was based on an expected seroprevalence of *T*. *cruzi* infection ranging from 30 to 50%; to minimize blood extraction rounds (which increase dropouts), and to provide conclusive serological results as early as possible. Two different ELISA tests and a third test as a tiebreaker were judged to ensure a high-quality diagnosis, whereas other screening tests registered at that time risked more false negative results and might lead to a greater loss of patients at a subsequent, confirmatory serosurvey.

Blood samples (1–3 mL) for DNA extraction and PCR were immediately mixed with an equal volume of guanidine hydrochloride 6 M, EDTA 0.2 M pH 8.0 buffer, and stored at 4°C [44]. The remainder of each blood sample was allowed to clot at ambient temperature, and serum was separated after centrifugation at 3,000 rpm for 15 min., allocated in labeled triplicate vials, transported in dry ice to the main laboratory at the end of each serosurvey, and preserved at -70°C until processing at the Laboratory of Eco-Epidemiology.

Each serum was tested for *T*. *cruzi* infection in duplicate by two ELISA tests using conventional (Chagatest, Wiener) and recombinant antigens (ELISA Rec V3.0, Wiener) according to manufacturer instructions. Their sensitivity and specificity were 100% and >99% (Chagatest) and 99.3% and 98.7–100% (ELISA Rec V3.0) according to the manufacturer; an international evaluation of commercial kits estimated Chagatest’s sensitivity and specificity at 98.8% and 99.6%, respectively [45]. Samples with an optical density deviation of 10% above the cut-off were considered positive, whereas those that were 10% below the cut-off were considered negative. Samples in the grey area between these limits were considered inconclusive and serological tests repeated. Sera that remained in the grey area after repetition and those with discordant results between both ELISAs were tested by an indirect immunofluorescence antibody test (Parasitest, Laboratorio IFI) with reported sensitivity of 100% and no cross-reaction with other prevalent diseases according to the manufacturer. Individuals positive by at least two different methods were considered seropositive for *T*. *cruzi*. For external quality control, a random sample of 30 sera were tested blindly at the national reference center for Chagas disease serodiagnosis (Instituto Nacional de Parasitología Dr M. Fatala Chaben); all were serologically concordant with previous results.

Detection of *T*. *cruzi* DNA by PCR provides evidence of early therapeutic failure [46–48]. For DNA extraction, guanidine-EDTA blood samples were heated in boiling water for 15 min. Prior to DNA extraction, 200 pg of an internal amplification control DNA was added to each 400 μL guanidine-EDTA aliquot [46]. Total DNA was purified using a commercial kit (DNeasy Blood & Tissue Kit, QIAGEN Sciences, Maryland, USA) according to manufacturer instructions barring use of proteinase K and buffer AL [46]. Purified DNA was eluted in 200 μL of distilled water and used as template for PCR amplification.

Total DNA samples were tested by a qualitative PCR assay targeted to the minicircles of the kinetoplast (kPCR) and by a quantitative PCR (qPCR) targeted to the nuclear satellite sequence as described elsewhere [44,49]. For qPCR, samples were run in duplicate using a commercial kit (SYBR GreenER qPCR SuperMix Universal, Invitrogen, Life Technologies, USA) at Instituto Nacional de Parasitología Dr. M. Fatala Chaben. Parasite DNA concentration was expressed as equivalent amounts of parasite DNA per ml (Pe/mL).

#### Treatment and follow-up

Local physicians communicated treatment benefits and eventual risks of ADRs to the patients and their parents or guardians at meetings held at the nearest rural school or healthcare post. A locally adapted plan for monitoring ADR and medication adherence was discussed and agreed upon (see below). The team coordinating these efforts included the head of the local healthcare primary system, research personnel (PS, IC), and a medical advisor from the National Chagas disease program (HF).

Treatment with benznidazole targeted all *T*. *cruzi*-seropositive individuals aged between 9 months and up to 18 years old, of any gender or ethnic group, who resided in the study area and signed the informed consent (inclusion criteria), and excluded pregnant or lactating women, individuals with renal or hepatic dysfunction or psychiatric problems or with severe or generalized disease or immunocompromised, and patients who reportedly had been treated with benznidazole or nifurtimox before. No upper limit for the number of treated individuals was fixed in advance.

Benznidazole (Radanil, Roche) was provided free of charge by the National Chagas disease program, and administered in two daily doses (5–8 mg/kg-day) during 60 days over April-July 2011. Tablets were fractioned by hospital pharmacist personnel to match individualized dosing regimens. Benznidazole doses and a weekly calendar were provided to each patient for each 15-day period to keep record of the doses taken daily and facilitate monitoring of medication adherence. No incentives were given to increase adherence. The senior author also completed a treatment diary to record the doses administered, its timing, and any symptoms or problems related to treatment.

The local physicians evaluated the patients’ clinical status at the healthcare posts or schools immediately before treatment (0 days posttreatment initiation, dpt) and at 10, 20–30 and 60 dpt, and recorded the results in an individual clinical history. Each clinical evaluation included at least one of the following exams: 1) semiologic exam: patients were canvased for previous or concurrent disease manifestations and ADRs, and a physical evaluation was conducted on each of the four occasions; 2) an electrocardiographic exam was conducted at 0 dpt and faxed to the Cardiology Service of the nearest reference hospital (in General San Martín, distant 100 km) for interpretation, and 3) clinical laboratory tests: blood samples were drawn by venipuncture at 0, 20–30 and 60 dpt to assess hematocrit and hemoglobin levels, platelets, white cell counts, alanine aminotransferase (ALT), aspartate aminotransferase (AST) and alkaline phosphatase (ALP), cholesterol, uremia, serum creatinine and serum proteins at the local hospital. Women in reproductive age were tested for pregnancy. A blood sample aliquot was kept for serological and molecular diagnosis as described above. Additional blood samples were collected at 180 dpt for serological and molecular diagnosis of *T*. *cruzi* infection. All sera collected at 0, 60 and 180 dpt were tested in parallel to assess whether specific antibody titers decayed relative to baseline values.

Monitoring of medication adherence and ADRs was conducted by local healthcare agents in five weekly appointments at the health post or at the patient’s home where they recorded the patient’s body weight and temperature, and the number of benznidazole pills remaining (S2 Table). The patients’ parents or guardians notified the healthcare agents the onset of any ADR sign or symptom, or in a few cases, they directly communicated with the coordination team by cell phone. All ADRs detected during drug administration (including type, date of onset and remission, duration, and specific treatment indicated) were recorded in each patient’s clinical history. ADRs were classified as dermatologic, neurologic or gastrointestinal, and their severity scored as mild, moderate or severe. Patients showing a severe or moderate, prolonged exanthema combined with fever were transported to the local hospital for medical evaluation. Temporary suspension or benznidazole dose reduction was prescribed to a few cases. Healthcare agents followed up the evolution of ambulatory patients until remission (S2 Table). Neither participants nor those administering the interventions or assessing the outcomes were blinded to study condition assignment.

### Data analysis

Treatment-related primary outcomes included treatment coverage and quality and therapeutic response. Treatment coverage was estimated as the percentage of seropositive patients up to 18 years of age at serodiagnosis that were treated with benznidazole relative to the number of seropositive patients in this age group who were eligible for treatment. Assessment of treatment quality was based on individual completion, medication adherence and ADR management. Adherence was evaluated at the periodic appointments through the percentage of benznidazole pills taken (i.e., number provided minus residual pills) relative to those provided for each specific time period, and then averaged over the treatment period. Patients with three or fewer pill counts and those who were withdrawn from or abandoned treatment were excluded from adherence estimates. Evaluation of ADR management included the percentage of patients who presented ≥1 ADR and were able to complete treatment; secondary outcomes included duration of the severest episode and proper notification of the event. Therapeutic response (i.e., treatment failure) was primarily evaluated through detection of *T*. *cruzi* DNA by kPCR and qPCR [3,13,47] in the subset of patients who completed the full treatment course and had ≥80% of medication adherence. Although conventional serodiagnosis was also performed, at least in the Gran Chaco region clearance of conventional anti-*T*. *cruzi* antibodies usually took several years even in young patients in the early chronic phase [12,16,17, 21]; therefore, we did not expect conventional serology would provide early evidence of seroconversion at 180 dpt. For additional comparisons, historical levels of treatment coverage with benznidazole or nifurtimox at the local hospital were estimated through a retrospective search of clinical histories of residents from the study area during the previous five years, and through householders’ reports.

We used Friedman’s two-way non-parametric analysis of variance to test for significant differences among repeated measurements of uremia, creatinine, AST, ALT and ALP performed before treatment and at 20 and 60 dpt, and Kendall’s *K* as an index of concordance. Fisher’s exact test or χ^2^ tests were used for investigating two by two contingency tables of independent data. Exact McNemar significance probabilities were calculated for paired data with small cell frequencies. The nominal level of statistical significance was set at a *P* value of 0.05. All tests were performed using Stata 12 [50].

## Results

**Fig 1:**
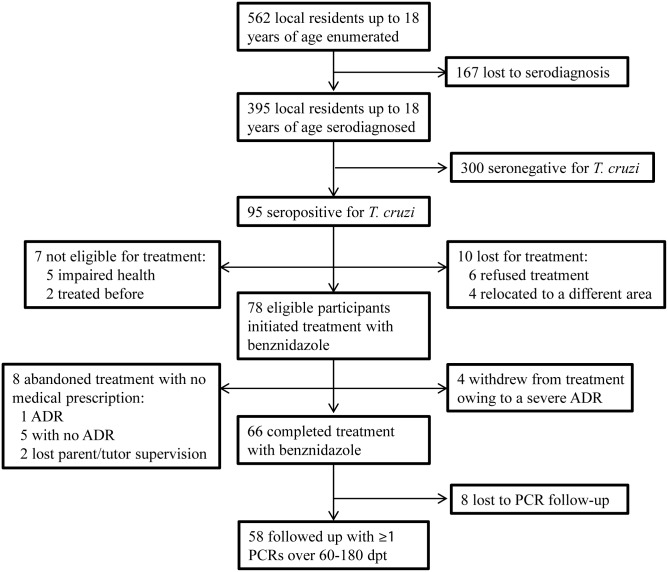
Flow chart of census, serodiagnosis, treatment with benznidazole and follow-up in 13 rural villages of Pampa del Indio, 2010–2011.

**Fig 2:**
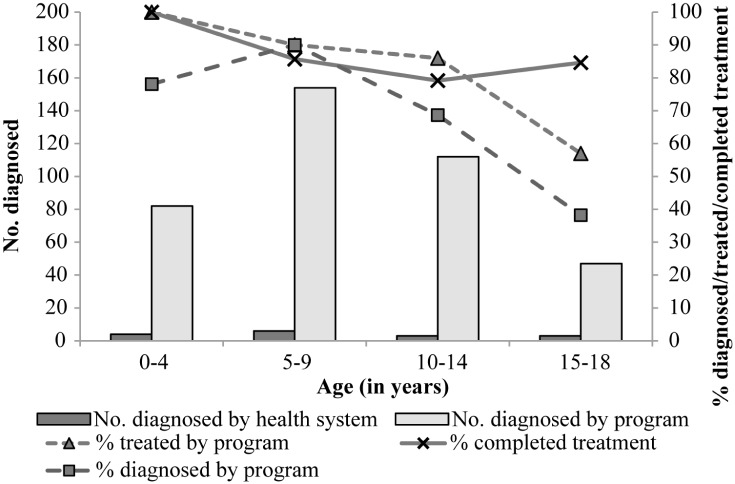
Coverage of serodiagnosis of *T*. *cruzi* infection and treatment with benznidazole according to patient’s age and source of interventions in Pampa del Indio.

The flow chart of the study population from census to follow-up is shown in Fig 1. The overall coverage of serodiagnosis was 70.3% (395 of 562) in children and adolescents aged up to 18 years old, with a peak in the age group 5–9 years old (Fig 2). The seroprevalence of *T*. *cruzi* infection in residents up to 18 years old was 24.1% (95 of 395). According to householders’ reports, the local health system had serologically examined for *T*. *cruzi* infection 16 (2.8%) local residents aged up to 18 years before the current intervention (Fig 2).

Ninety-five seropositive individuals were screened for eligibility (Fig 1). Reasons for lack of participation included refusal to initiate treatment, household emigration from the study area, impaired health conditions, and a previous treatment with benznidazole or nifurtimox. Treatment with benznidazole was initiated by all 78 eligible participants (Fig 1). Treatment coverage decreased with increasing age (Fig 2). Crude treatment coverage among all identified seropositive residents was 82% (78 of 95). The mean (±SD) age at treatment was 11.0±4.0 years old (range, 3–19), and 36 (46%) treated patients were females.

All seropositive children who initiated treatment were asymptomatic and displayed the indeterminate form of Chagas disease at baseline except one (of 71 patients examined by electrocardiography) with a congenital arrhythmia unrelated to Chagas. Hematocrit, hemoglobin, platelets, white cell counts and liver enzymes usually were within normal limits, except one case of anemia and 27 with eosinophilia (≥10%); these laboratory results did not prevent the initiation of treatment. Friedman’s test showed statistically significant changes over 0, 20 and 60 dpt in creatinine levels (*P* = 0.026, *K* = 0.502), AST (*P* = 0.012, *K* = 0.485), ALP (*P* < 0.001, *K* = 0.641), and marginally significant changes in uremia (*P* = 0.077, *K* = 0.420) and ALT (*P* = 0.082, *K* = 0.421). Although most laboratory tests remained within normal limits during treatment, three patients presented a ≥2× increase of ALP at 20 dpt coinciding with ADR episodes whereas other three showed elevated levels of ALT and ADRs. Eleven patients had a ≥2× increase of ALP at 60 dpt, but most of them had no ADR. No patient interrupted treatment due to laboratory abnormalities.

Treatment was completed by 66 (85%) of the 78 patients enrolled in the study (Fig 1). Completion rates decreased from 100% among children <5 years to 85% among young people aged 15–19 years (Fig 2). Individuals who did not complete treatment tended to be older (12.4±6.0 years, range 9–17) and had a balanced gender distribution (50%). Medication adherence across patients who completed the full treatment course averaged 97% (range, 80–100%) (Table 1). Among the 12 patients who did not complete treatment, four took medication for 25–28 days (range of adherence, 70–100%); other four took it for 4–17 days, and no data were provided by four patients. For comparison, the local health system had treated with benznidazole only two *T*. *cruzi*-seropositive children residing in the study area over the previous five years.

**Table 1:** Frequency, severity and duration of the severest episode of any adverse drug-related reaction (ADR), treatment completion rates and medication adherence in 76 patients treated with benznidazole in Pampa del Indio, excluding two patients with an early dropout. Nd, no data.

Type of ADR	Severity	No. of patients (%)	Mean duration of severest^#^ episode (days)	Medication adherence (%)^##^	Treatment completion
None	None	52 (69)	0	97	48 (92)
Exanthema	Mild	10 (13)*	2.1	96	9 (90)
	Moderate	5 (7)**	5.6	100	4 (80)
	Severe	3 (4)***	2.7	100	0 (0)
	Unknown	3 (4)	4.0	82	2 (67)
Dizziness	Mild	1 (1)	Nd	100	1 (100)
Headache	Moderate	1 (1)	Nd	100	1 (100)
Gastrointestinal	Mild	1 (1)	Nd	94	1 (100)
Total		76	3.4	97	66 (87)

* One combined with mild myalgia and severe arthralgia.

** One combined with mild headache.

*** One combined with mild fever.

^#^ Computed among participants displaying ≥1ADR episode.

^##^ Computed among participants who completed the full treatment course.

A total of 24 (32%; 95% CI: 21–43%) patients presented ≥1ADR (Table 1), and 20 (83%) reported it through the agreed mechanisms. Table 1 only includes the severest presentation for the six patients with 2 or 3 ADR episodes. The mean age of patients showing ≥1 ADR (12.0 years, 95% CI: 10.3–13.7) was not significantly different from that of patients showing no ADR (10.5 years, 95% CI: 9.4–11.6). On average, the first ADR appeared at 13.3 dpt initiation (95% CI: 10.8–15.8; range, 4–29 dpt).

Thirty-eight ADR episodes were recorded, including 22 mild, 9 moderate and 4 severe presentations, and 3 dermatological ADRs whose severity could not be established because of late reporting (Table 1). Twenty-one (88%) patients with ≥1 ADR displayed maculopapular exanthema, alone or combined with mild headaches or an increasingly severe arthralgia/myalgia. No Lyell or Stevens-Johnson syndromes and polyneuritis were recorded. Mild and moderate ADRs were managed in an out-patient basis; physicians evaluated the patients and eventually administered antihistamines, paracetamol and ibuprofen. Temporary dose reductions were indicated to four patients. Benznidazole was temporarily suspended for an average of 4 days in six patients showing mild or moderate exanthema and mild to severe arthralgia/myalgia or moderate headaches. Treatment withdrawal (followed by short-term hospitalization and administration of corticoesteroids and antihistamines) was prescribed to four patients displaying repeat ADR episodes that did not respond to symptomatic medication (age range, 6–17 years): three had a severe exanthema (one with fever) and one a moderate, prolonged exanthema and headaches. Duration of the severest ADR episode took on average 3.4 days (range, 1–7 days). Medication adherence varied very little among ADR types and levels.

Eighteen (75%) patients with ≥1ADR were able to complete treatment. Treatment completion rates significantly decreased with increasing ADR severity from 90–92% among patients with either no ADR or mild exanthema (including headaches and dizziness) to 0% among the three patients with a severe exanthema (Fisher’s exact test, *P* = 0.011), after pooling no or mild ADRs versus moderate or severe ADRs to avoid small cell frequencies (Table 1). One patient who failed to report an ADR (exanthema) abandoned treatment without medical indication and prompted other four family members under treatment to do so despite they had no ADR. Among patients having another household member under treatment, the relative odds of presenting at least one ADR was significantly and positively associated with having another household member with an ADR (OR = 2.57; 95% CI: 1.02–7.28; exact McNemar significance, *P* = 0.043).

The therapeutic response to treatment as determined by kPCR and qPCR is shown in Table 2. Before treatment, 40 (65%) patients were co-positive by both PCRs, 15 (24%) were co-negative, and discordant results occurred in 7 (11%) qPCR-positive and kPCR-negative patients; the performance of both PCRs differed significantly (exact McNemar significance, *P* = 0.016). During treatment, only one (2%) patient was kPCR-positive and qPCR-negative whereas the remainder was co-negative. Immediately after treatment at 60 dpt, 56 (97%) patients were co-negative, 1 (2%) was kPCR-negative and qPCR-positive, and 1 (2%) showed the reverse discordant pattern. This patient`s parasite burden decreased from 20 Pe/mL before treatment to 0.44 at 60 dpt and was negative thereafter. At 180 dpt, the only patient who was co-positive had shown *T*. *cruzi* DNA amplification at 20 dpt by kPCR (i.e., treatment failure) and refused the offer for new treatment with nifurtimox. The relative frequency of kPCR- or qPCR-positive results before treatment highly significantly declined by 180 dpt (exact McNemar significance, *P* < 0.0001). Median parasite load among kPCR- or qPCR-positive patients before treatment drastically fell from 1.4 Pe/mL to undetectable levels at 20 dpt and thereafter remained much lower than before treatment among the three patients positive by either PCR (Table 2). Of the 46 patients who were qPCR-positive before treatment, only two were positive at 60 (subsequently negative) or 180 dpt; the remainder was two (42) or three (38) times negative over 20–180 dpt. Of 16 patients initially qPCR-negative, none was positive over 20–180 dpt.

**Table 2:** kPCR and qPCR qualitative outcomes and parasite burden before and at 20–180 days posttreatment with benznidazole in *T*. *cruzi*-seropositive children up to 18 years old, Pampa del Indio.

Time posttreatment (days)	kPCR	qPCR		Parasite burden (Pe/mL)*	
		Positive	Negative	Median	Q1-Q3
0	Positive	40	0	1.4	0.1–4.4
	Negative	7	15		
20	Positive	0	1	0	
	Negative	0	54		
60	Positive	0	1	0.3	
	Negative	1	56		
180	Positive	1	0	0.7	
	Negative	0	48		

* Among kPCR-positive or qPCR-positive patients.

The treated patients who participated in the serological follow-up at 60 or 180 dpt showed virtually no decay in paired optical densities by the Chagatest ELISA between 0 and 60 dpt (mean percent absolute reduction, 5.5%; 95% confidence interval, CI, 1.3–9.8) or between 0 and 180 dpt (mean, 0.5%; CI, -1.6–1.7%). Only one patient had a drop in optical density levels below the Chagatest cutoff value. Using recombinant ELISA, mean percent absolute reductions between 0 and 60 dpt (6.6%; CI, 2.9–10.4%) and between 0 and 180 dpt (mean, 7.1%; CI, 2.3–11.8%) were slight at most, with only one patient having a >60% drop in optical densities.

## Discussion

Our study demonstrates that diagnosis and treatment of *T*. *cruzi* infection in remote, impoverished rural areas can be effectively addressed through strengthened primary healthcare attention and broad social participation combined with adequate external support. This strategy took advantage of locally available resources; secured high levels of diagnostic and treatment coverage and medication adherence, effectively managed ADRs at village level under carefully administered protocols, and provided early evidence of positive therapeutic responses except in one case. Although the efficacy of benznidazole has been firmly established in hospitals or specialty clinics [15,16], especially among young patients, the issues of treatment access, adherence and effectiveness in remote, hyperendemic rural settings have received little attention.

Broad social participation implied a multi-stakeholder agreement that included the affected communities and other sectors. The community workshops were essential to raise awareness of Chagas disease characteristics and consequences, diagnostic and treatment opportunities, and provided an appropriate context to reach an agreement on intervention details adapted to local circumstances. A key output was the two-sided commitment for implementing interventions and building of mutual trust. Compliance with dates for the expected return of serological results (unlike in other health interventions reported by householders) facilitated further community involvement with treatment and follow-up activities.

The local health system faced critical constraints (e.g., medical personnel and vehicle) at the time of the interventions. These facts, combined with considerable distances between rural villages and the local hospital, led to a centralized health service delivery model to which the rural population had poor access. The initial workshop outputs identified that most activities had to be conducted at the residents’ villages. Strengthening the capacity of rural healthcare agents and medical personnel was essential: the former undertook monitoring of medication adherence and ADRs, whereas the *in situ* participation of physicians was restricted to treatment indication, clinical exams and improved ADR management. In remote areas of Africa, transference of treatment initiation and monitoring of HIV patients to local nurses improved the access to and quality of health care and cost-effectiveness of integrated interventions [51,52]. Benznidazole was available at the time of our program and timely provision was carefully planned, but these usually are major issues elsewhere. As a direct repercussion of the current program, spontaneous treatment demand at the local hospital increased substantially over subsequent years and included adult patients.

The degree of diagnostic coverage attained among rural residents up to 18 years old was substantial (70.3%) and paralleled or exceeded levels recorded in rural communities of the Argentine Chaco [6,39] and by the local health system. The enhanced diagnostic coverage was partly related to the prospects of receiving treatment and eventual welfare benefits derived from being seropositive for *T*. *cruzi*.

Treatment completion rates were likely enhanced through the contribution of dedicated staff supervising the onset of benznidazole administration and ADR follow-up; no incentives for treatment initiation, completion and follow-up were given. Medication adherence was very high and similar to the levels recorded in urban or rural populations through other approaches [19,21,22]. The healthcare agent- and parent-based monitoring system was key to early identify patients showing ADRs and breaches in adherence. Likewise other benznidazole trials in children [19,21], most ADRs occurred during the first two weeks after onset of treatment; close patient monitoring during this period is therefore indicated. No significant age-related increase of the occurrence of an ADR was detected, unlike in studies covering a wider age range [20,26]. The significant household aggregation of ADRs suggests putative genetic or environmental factors acting on a familial level that may possibly modify the bioavailability of or response to benznidazole. Elevated levels of ALT or ALP during or immediately after benznidazole treatment occurred in several patients, but they were not as important as to interrupt treatment (rarely exceeded baseline levels by 3×) nor were laboratory abnormalities associated with the occurrence of an ADR.

The most frequent benznidazole-related ADRs were dermatological (usually of mild or moderate severity) as in several other studies and locations, although their frequency tends be quite variable [12,19,21,22]. Most mild or moderate ADRs were successfully managed with symptomatic medication, dose reduction and temporary suspension of benznidazole intake, whereas the four cases with severe ADRs required treatment withdrawal, short-term hospitalization and symptomatic medication. Treatment completion was significantly and inversely related to ADR severity, again supporting the need of close monitoring and adequate ADR management as a means of increasing completion rates and preventing treatment abandonment in other family members.

qPCR was significantly more sensitive than kPCR to detect *T*. *cruzi* DNA before treatment and evidenced the rapid fall of circulating parasites as early as three weeks after onset of treatment [20,48]. Evidence from several trials supports that qPCR is an early marker of treatment failure [13,31,46–48]. The proportion of *T*. *cruzi*-seropositive patients initially qPCR-positive (65%) was very close to the range recorded in several other studies targeting the same age group and early chronic infections [18,53,54]. Moreover, all children [22] and the great majority of adult patients [13] treated with benznidazole for 60 days remained qPCR-negative over 1–1.5 years of follow-up after effective treatment, hence suggesting that under such conditions recurrence of parasitemia appears to be rare but other patterns have been reported [e.g., 18]. In our study, the only patient with persistently positive kPCR or qPCR tests (before and at 20 and 180 dpt) was a 12-year-old child with rather limited adherence (80%) who had no travel history outside the study area and resided in a non-infested house (i.e., a treatment failure). However, a sizable fraction of adult individuals under a partial treatment course with benznidazole have shown positive therapeutic responses [55]. A second patient with very much reduced parasite burden from 0 to 60 dpt (but still qPCR-positive) was subsequently qPCR-negative, as recorded by others [46]. Most important, all patients but two were subsequently qPCR-negative on two or three occasions over 20–180 dpt. Although we cannot exclude whether longer follow-up times would allow some of the molecular tests to yield a positive result, the individual time patterns of qPCR including two or three negative results in a row are more compatible with a process of parasite clearance in young patients in the early chronic phase of infection [cf 14.17].

Intensified vector control and systematic surveillance was deemed a prerequisite for launching the diagnosis-and-treatment program because fast house reinfestation after insecticide spraying frequently causes new human infections in rural areas of the Gran Chaco [5,8,10] and elsewhere [18,56]. In the face of moderate pyrethroid resistance, suppressing house infestations demanded recurrent insecticide applications [40] and delayed treatment activities. However, this prolonged process laid the foundations for subsequent participatory activities; averted the chance of new vector-mediated human infections, and determined that child infections most likely had been acquired at least 3–4 years before treatment (i.e., early chronic infections).

Our study had several limitations. Generalizability of the current strategy to other areas depends on the existence of a primary healthcare service. Medication adherence was measured through pill counts in the absence of a practical assay for establishing serum benznidazole levels at the study setting. In the absence of a well-established biomarker of early cure [26,47], the time-limited follow-up of patients up to 180 dpt precluded us from using seroconversion to a negative status as a primary endpoint. Seroconversion usually takes several years even in young residents from the Gran Chaco in the early chronic phase [12,16,17,21], depending on the time span between primary infection and treatment and other ill-defined determinants, which explains the nearly stable pattern of ELISA optical densities before and after treatment. We note, however, that results elsewhere in central Brazil [14,15], Guatemala and Honduras [21] and Colombia [18] showed quite diverse patterns of conventional antibody clearance rates in response to benznidazole treatment in young patients <15 years old. Losses to follow-up via PCRs at 180 dpt included 17 (26%) of 66 patients who completed treatment and may bias estimates of treatment effect size. Cohort attrition over the follow-up is typical in trials conducted in rural locations, more so in remote areas with migrant populations. Whether *T*. *cruzi*-infected children treated with benznidazole will show improved long-term clinical outcomes is a crucial question that merits further research. Conclusions from this study may not be extrapolated to adult patients who have more frequent and severe ADRs with a different time pattern [22].

Our study links community participation to a health outcome improvement [57,58]. Community participation in remote rural settings is essential for treatment programs [32]. Increasing access to high-quality serodiagnosis and treatment of marginalized rural populations, combined with effective vector control and surveillance in the affected regions, is ethically imperative.

## Supporting information

S1 Table Strengths, opportunities and threats identified in participatory workshops including community, health and research personnel. Pampa del Indio, Chaco, 2011.
(DOCX)

S2 Table Roles assumed by participants across the various phases of the intervention program. Pampa del Indio, Chaco, 2010–2011.
(DOCX)

S3 Table Individual patient data including serodiagnosis, treatment, adverse reactions, and PCR outcomes. Pampa del Indio, Chaco, 2010–2011.
(RAR)

S1 Text TREND statement checklist for observational studies. (PDF)

S2 Text Treatment protocol. (DOCX)
