# Supplementary material for: Improving access to Chagas disease diagnosis and etiologic treatment in remote rural communities of the Argentine Chaco through strengthened primary health care and broad social participation
Source: PLoS Negl Trop Dis. 2017 Feb 13;11(2):e0005336. doi: 10.1371/journal.pntd.0005336 (PMC5325580; doi:10.1371/journal.pntd.0005336)
Supplement: S1 Table — Pampa del Indio, Chaco, 2011. (DOCX) [file pntd.0005336.s001.docx]

S1 Table: Strengths, opportunities and threats identified in participatory workshops including community, health and research personnel in Pampa del Indio, 2011.

| Stakeholder | Strengths and opportunities | Threats |
| --- | --- | --- |
| Village/  community | Rural school system  Social organization  Radio communication  Cell phone signal active in some rural sections  High school attendance | Scarce transportation means  Long distance to hospital  Limited access via dirt roads  Scarce economic resources  Frustration with mass serosurveys |
| Rural health posts | Close links to villagers  Within reasonable distance from patients’ homes | Poor building conditions  Scarce first-aid means and equipment  No communication means with the hospital  No physician attendance  Large areas to service  Health agents residing elsewhere  No experience with *T. cruzi* treatment |
| Hospital | Experience in Chagas etiologic treatment  Complementary healthcare services available  Medication for ADR management available | No experience with ADR management  Overburdened medical personnel  Limited transportation means  Reduced laboratory capacity  No ELISA reader  Provision of benznidazole irregular or with long delays |
