# Supplementary material for: Improving access to Chagas disease diagnosis and etiologic treatment in remote rural communities of the Argentine Chaco through strengthened primary health care and broad social participation
Source: PLoS Negl Trop Dis. 2017 Feb 13;11(2):e0005336. doi: 10.1371/journal.pntd.0005336 (PMC5325580; doi:10.1371/journal.pntd.0005336)
Supplement: S2 Table — Pampa del Indio, Chaco, 2010–2011. (DOCX) [file pntd.0005336.s002.docx]

S2 Table: Roles assumed by participants across the various phases of the diagnosis-and-treatment program in Pampa del Indio, 2010-2011.

| Stakeholder | Activity | Frequency |
| --- | --- | --- |
| Healthcare agents | Explanation of informed consent | Before blood extraction and before treatment |
|  | Serosurvey support and delivery of diagnostic results | Between 30 and 120 days after blood extraction |
|  | Delivery of medication and monitoring of treatment adherence | Weekly during the first 15 dpt, and every 10 days until final completion |
|  | ADR detection | During drug intake |
|  | Monitoring of ADR evolution | During drug intake |
| Biochemists | Biochemical assays | At 0, 20-30 and 60 dpt |
| Local physicians | Clinical exams | At 0, 20-30 and 60 dpt |
|  | ADR management | During drug intake |
| Chagas program | Delivery of benznidazole | Before treatment onset |
| Hospital pharmacy | Dose preparation | Biweekly during drug intake |
| Research group | Collection, separation, transportation and preservation of blood samples | During blood extraction at 0, 20-30, 60 and 180 dpt |
|  | Serodiagnosis | Between 30 and 120 days after blood extraction |
|  | Molecular diagnosis | After blood extractions |
| Coordination team | General coordination and and supervision of procedures | During drug intake |
|  | Medical counseling and facilitation |  |
|  |  |  |

dpt, days posttreatment initiation.
