# Supplementary material for: Improving access to Chagas disease diagnosis and etiologic treatment in remote rural communities of the Argentine Chaco through strengthened primary health care and broad social participation
Source: PLoS Negl Trop Dis. 2017 Feb 13;11(2):e0005336. doi: 10.1371/journal.pntd.0005336 (PMC5325580; doi:10.1371/journal.pntd.0005336)
Supplement: S2 Text — (DOCX) [file pntd.0005336.s005.docx]

**Text S2. Treatment protocol.**

# Improving access to Chagas disease diagnosis and etiologic treatment in remote rural communities of the Argentine Chaco

## Principal Investigators

Dr. Sartor, Paula

Dr. Cardinal, Marta Victoria

Dr. Gürtler Ricardo Esteban

Universidad de Buenos Aires. Consejo Nacional de Investigaciones Científicas y Técnicas. Instituto de Ecología, Genética y Evolución de Buenos Aires (IEGEBA), Facultad de Ciencias Exactas y Naturales, Buenos Aires, Argentina.

Dr. Freilij, Héctor

Programa Nacional de Chagas, Ministerio de Salud de la Nación, Buenos Aires, Argentina.

## Co-Investigators

Dr. Bua, Jacqueline,

Instituto Nacional de Parasitología "Dr. M. Fatala Chaben", ANLIS C.G. Malbrán, Buenos Aires, Argentina,

Dr. Colaiani, Ivana

Hospital Santojanni, Buenos Aires, Argentina,

# INTRODUCTION

##

## Background

Chagas disease ranks among the main neglected tropical diseases (NTDs) in Latin America and the Caribbean [1]. *Trypanosoma cruzi*, its etiologic agent, induces heart and digestive disease and reduces life expectancy in approximately 30-40% of the infected people [2,3]. The parasite infects 6-9 million people, the majority of which primarily were rural residents living in poverty with little access to healthcare services [4]. A well-known hotspot of Chagas disease and other NTDs is the Gran Chaco ecoregion which mainly extends over sections of Argentina, Bolivia, and Paraguay [5].

The two drugs (nifurtimox and benznidazole) registered for treatment of human infection with *T. cruzi* since the late 1960s and early 1970s were shown to be especially effective in young age groups during the acute and early chronic phase regardless of transmission mode [3,6-10]. Both drugs may cause adverse drug-related reactions (ADR) of various types, frequency and severity [11-14]. Less than 1% of patients infected with *T. cruzi* have access to parasiticidal treatment [15]. The challenge of how to address diagnosis and treatment of *T. cruzi* infection in resource-poor, remote rural settings through primary health care has yet to be developed and program effectiveness documented to meet the challenge of treating the sizable population of infected rural residents and correct health inequities.

**OBJECTIVE**

To develop, implement and test a strategy based on strengthened primary healthcare attention and broad social participation with the goals of increasing access to diagnosis and treatment of human *T. cruzi* infection in sparsely populated rural sections of Pampa del Indio municipality.

# study design

## Study type

Non-randomized prospective study in children residing in a well-defined rural endemic area.

## Population

### Selection criteria

Children aged between 9 months and 18 years old resident of Pampa del Indio, (rural) Area I as defined in Gurevitz et al. 2011, who were seropositive for *T. cruzi*.

### Inclusion criteria

- Children of any gender or ethnic group, aged between 9 months and 18 years old, residents of Pampa del Indio Area I, seropositive for *T. cruzi* by at least two different techniques and eligible for treatment with benznidazole as per this treatment protocol.
  - Serodiagnostic criteria: At least 2 positive serological tests for *T. cruzi* infection using conventional (Chagatest, Wiener) and recombinant antigens (ELISA Rec V3.0, Wiener), and for serologically discordant sera indirect immunofluorescence antibody assay (Parasitest, Laboratorio IFI).
- Informed consent signed by the parent or guardian and consent of the patient. For community meetings including indigenous residents, explanations will be translated by an indigenous healthcare agent or by an appointed indigenous community member and consent will be obtained collectively and individually.

### Exclusion Criteria

- Pregnant or lactating women.
- Individuals with renal or hepatic dysfunction or psychiatric problems or with severe or generalized disease or immunocompromised.
- Patients who reportedly had been treated with benznidazole or nifurtimox before.

### Criteria for withdrawal from the study

- Severe adverse reaction potentially attributable to benznidazole or not.
- Any situation that may put the safety of the patient at risk, according to the judgment of attending physicians.

# research plan

## Study area

## The intervention will be conducted in Pampa del Indio (25°55’S 56°58’W), Chaco, Argentina. The study area (denominated Area 1) included 353 houses and a few public buildings grouped in 13 rural villages distributed over a 450 km^2^ section as described in Gurevitz et al. (2011). The study area was inhabited by 1,187 people in 2007. The only existing medical facility is a first-level public hospital with four physicians; the primary healthcare system had 5 posts distributed across the study area, and there were 8 primary schools.

All inhabited house compounds were sprayed with pyrethroid insecticides in November-December 2007. All houses found to be reinfested over 2008-10 were selectively re-sprayed with insecticides after each periodic survey. House infestation at the time of the planned human serosurveys (September 2010-March 2011) was <1% and mainly occurred in peridomestic structures; none of the bugs collected were infected with *T. cruzi* as determined by microscopic analysis of feces at 400×.

## General overview of the study

The study includes five successive phases: preparatory, participatory planning, capacity strengthening of local health personnel, diagnostic surveys, and treatment and follow-up, all of which were deemed to be completed within 1.5 years.

Preliminary meetings with local authorities, health personnel, rural school teachers, and community leaders will be conducted to inform them of the planned intervention program and request their feedback. Community workshops will be conducted to introduce the research team, communicate the program’s objectives and phases, identify local capacities and weaknesses linked to healthcare activities, and establish a permanent channel of communication with local referents. Householders of each target village within Area 1 of Pampa del Indio will be convened by radio broadcasts and written messages to schoolchildren’s parents at each rural school. Capacity strengthening of health personnel will be achieved over three meetings whose ultimate goal is to define more precisely the roles of rural healthcare agents and coordination team members, and to adapt field activities to the detected constraints.

## Diagnostic surveys

*Serosurveys.* Residents from all the study villages will be summoned to the nearest healthcare post or school for blood sample extraction. Blood samples will be drawn by venipuncture from all residents older than one year of age (3 mL from children aged 1-2 years old, and 5-7 mL from older patients), and capillary blood taken from infants aged 9-12 months by fingerprick or heal prick. Venipuncture is preferred to collecting whole blood on filter paper or using other methods because of the large seroprevalence of *T. cruzi* infection expected (30 to 50%); to minimize blood extraction rounds (which increase dropouts), and to provide conclusive serological results as early as possible and progress to the treatment phase. Diagnostic surveys and patient recruitment will be carried out by Dr. Paula Sartor and Dr. Ivana Colaiani. The diagnostic surveys will be conducted over 3-5 months at local schools or health posts.

Each serum will be tested for *T. cruzi* infection in duplicate by two ELISA tests using conventional (Chagatest, Wiener) and recombinant antigens (ELISA Rec V3.0, Wiener) according to manufacturer instructions, at the Laboratory of Eco-Epidemiology in Buenos Aires. Serologically discordant samples will be tested by an indirect immunofluorescence assay (Parasitest, Laboratorio IFI). Individuals positive by at least two different methods will be considered seropositive for *T. cruzi*. For external quality control, a random sample of 30 sera will be tested blindly at the Instituto Nacional de Parasitología Dr M. Fatala Chaben.

*Molecular diagnosis.* Detection of *T. cruzi* DNA will be conducted using a qualitative PCR assay targeted to the minicircles of the kinetoplast (kPCR) and by a quantitative PCR (qPCR) targeted to the nuclear satellite sequence [44,49]. For qPCR, samples will be run in duplicate using a commercial kit (SYBR GreenER qPCR SuperMix Universal, Invitrogen, Life Technologies, USA) at Instituto Nacional de Parasitología Dr. M. Fatala Chaben. Parasite DNA concentration will be expressed as equivalent amounts of parasite DNA per ml (Pe/mL).

## Patient recruitment

Patients found to be seropositive for *T. cruzi* and their parents or guardians will be summoned for a meeting conducted at the nearest rural school or healthcare post. Local physicians will communicate treatment benefits and eventual risks of ADRs to the patients. A locally adapted plan for monitoring ADR and medication adherence will be discussed and agreed upon.

Prior to the onset of treatment with benznidazole, the attending physicians will evaluate the patients’ clinical status at the healthcare posts or schools immediately before treatment (0 days posttreatment initiation, dpt) and record the results in an individual clinical history. Each clinical evaluation will include a semiologic exam, an electrocardiographic exam, and clinical laboratory tests to assess hematocrit and hemoglobin levels, platelets, white cell counts, alanine aminotransferase, aspartate aminotransferase and alkaline phosphatase, cholesterol, uremia, serum creatinine and serum proteins at the local hospital. Women in reproductive age will be tested for pregnancy. A blood sample aliquot will be kept for serological and molecular diagnosis as described above.

## Treatment

Benznidazole (Radanil^®^, Roche) will be provided free of charge by the National Chagas disease program, and administered in two daily doses (5-8 mg/kg-day) during 60 days over April-July 2011 by parents or guardians in an outpatient setting. Tablets will be fractioned by an hospital pharmacist to match individualized dosing regimens. Benznidazole doses and a weekly calendar will be provided to each patient, parent or guardian for each 15-day period to keep record of the doses taken daily and facilitate monitoring of medication adherence.

## Follow up

Monitoring of medication adherence and ADRs will be conducted by local healthcare agents in five weekly appointments at the health post or at the patient’s home where they will record the patient’s body weight and temperature, and the number of benznidazole pills remaining. The patients’ parents or guardians will notify the healthcare agents the onset of any adverse drug-related reaction, sign or symptom, or may directly communicate with the coordination team by cell phone. The research team will keep a treatment diary to record the doses administered, its timing, and any symptoms or problems related to treatment.

All ADRs detected during drug administration (i.e., type, date of onset and remission, duration, and specific treatment indicated) will be recorded in each patient’s clinical history. Patients showing a severe or moderate, prolonged exanthema combined with fever will be transported to the local hospital for medical evaluation. Temporary suspension or benznidazole dose reduction or treatment with corticoesteroids or antihistamines may be prescribed to patients with mild or moderate reactions. Healthcare agents will follow up the evolution of ambulatory patients until remission.

The local physicians will re-evaluate the patients’ clinical status at the healthcare posts or schools 10, 20-30 and 60 dpt, and add these results to each individual clinical history. Each clinical evaluation will include a semiologic exam and clinical laboratory tests at 20-30 and 60 dpt to assess the same variables as determined at baseline. A blood sample aliquot will be kept for serological and molecular diagnosis as described above. Additional blood samples will be collected at 180 dpt for serological and molecular diagnosis of *T. cruzi* infection. All sera collected at 0, 60 and 180 dpt will be tested in parallel to assess any decay in specific antibody titers relative to baseline values.

# Data Analysis and statistical methods

Treatment-related primary outcomes included treatment coverage (i.e., percentage of seropositive patients up to 18 years of age at serodiagnosis that were treated with benznidazole relative to the number of seropositive patients in this age group who were eligible for treatment); treatment quality (based on individual completion, medication adherence – as determined by the average percentage of benznidazole pills taken relative to those provided for each specific time period- and ADR management --percentage of patients who presented ≥1 ADR and were able to complete treatment), and therapeutic response (detection of *T. cruzi* DNA by kPCR and qPCR in patients who completed the full treatment course and had ≥80% of medication adherence). Patients with three or fewer pill counts and those withdrawn from or who abandon treatment will be excluded from adherence estimates. The therapeutic response to treatment will be essentially measured by the proportion of patients who were qPCR-negative at 180 dpt among those who had been qPCR-positive before treatment.

Statistical analysis will include Friedman’s two-way non-parametric analysis of variance for testing differences among repeated measurements of biochemical variables before treatment and at 20 and 60 dpt; Kendall’s *K* as an index of concordance; Fisher’s exact test or χ^2^ tests for investigating two by two contingency tables of independent data, and exact McNemar significance probabilities for paired data with small cell frequencies. The nominal level of statistical significance is set at a *P* value of 0.05. All tests will be performed using Stata 12.

# ethical considerations

Informed written consents will be taken as detailed above. All clinical investigations will be conducted according to the principles expressed in the Declaration of Helsinki and the regulations of the Argentine Ministry of Health and the Ministry of Health of Chaco Province.

# data confidentiality

Patient data confidentiality will be strictly ensured. Samples will be identified by alphanumeric codes. Patient data will be stored in files and computers to which only the principal investigators and co-investigators will have access.

# bibliography

1. Hotez PJ, Alvarado M, Basañez MG, Bolliger I, Bourne R, et al. (2014) The global burden of disease study 2010: interpretation and implications for the neglected tropical diseases. PLoS Negl Trop Dis 8: e2865.
2. Rassi A Jr, Rassi A, Marin-Neto JA (2010) Chagas disease. Lancet 375: 1388-1402.
3. Lescure FX, Le Loup G, Freilij H, Develoux M, Paris L, Brutus L, Pialoux G (2010) Chagas disease: changes in knowledge and management. Lancet Infect Dis 10: 556-570.
4. Hotez PJ (2014) Ten global "hotspots" for the neglected tropical diseases. PLoS Negl Trop Dis 8: e2496.
5. Gürtler RE (2009) Sustainability of vector control strategies in the Gran Chaco Region: current challenges and possible approaches. Mem Inst Oswaldo Cruz 104: 52-59.
6. Molina I, Gómez i Prat J, Salvador F, Treviño B, Sulleiro E, et al. (2014) Randomized trial of posaconazole and benznidazole for chronic Chagas’ disease. N Engl J Med 370: 1899–1908. http://dx.doi.org/10.1056/NEJMoa1313122.
7. de Andrade AL, Zicker F, de Oliveira RM, Almeida Silva S, Luquetti A, et al. (1996) Randomised trial of efficacy of benznidazole in treatment of early *Trypanosoma cruzi* infection. Lancet 348: 1407-1413.
8. de Andrade AL, Martelli CM, Oliveira RM, Silva SA, Aires AI, et al. (2004) Short report: benznidazole efficacy among *Trypanosoma cruzi*-infected adolescents after a six-year follow-up. Am J Trop Med Hyg 71: 594-597.
9. Sosa Estani S, Segura EL, Ruiz AM, Velazquez E, Porcel BM, et al. (1998) Efficacy of chemotherapy with benznidazole in children in the indeterminate phase of Chagas' disease. Am J Trop Med Hyg 59: 526-529.
10. Flores-Chavez M, Bosseno MF, Bastrenta B, Dalenz JL, Hontebeyrie M, et al. (2006) Polymerase chain reaction detection and serologic follow-up after treatment with benznidazole in Bolivian children infected with a natural mixture of *Trypanosoma cruzi* I and II. Am J Trop Med Hyg 75: 497-501.
11. Bianchi F, Cucunubá Z, Guhl F, González NL, Freilij H, et al. (2015) Follow-up of an asymptomatic Chagas disease population of children after treatment with nifurtimox (Lampit) in a sylvatic endemic transmission area of Colombia. PLoS Negl Trop Dis 9: e0003465.
12. Altcheh J, Moscatelli G, Moroni S, Garcia-Bournissen F, Freilij H (2011) Adverse events after the use of benznidazole in infants and children with Chagas disease. Pediatrics 127: 212-218.
13. Altcheh J, Moscatelli G, Mastrantonio G, Moroni S, Giglio N, et al. (2014) Population pharmacokinetic study of benznidazole in pediatric Chagas disease suggests efficacy despite lower plasma concentrations than in adults. PLoS Negl Trop Dis 8: e2907.
14. Yun O, Lima MA, Ellman T, Chambi W, Castillo S, et al. (2009) Feasibility, drug safety, and effectiveness of etiological treatment programs for Chagas disease in Honduras, Guatemala, and Bolivia: 10-year experience of Médecins Sans Frontières. PLoS Negl Trop Dis 3: e488.
15. Viotti R, Vigliano C, Lococo B, Alvarez MG, Petti M, et al. (2009) Side effects of benznidazole as treatment in chronic Chagas disease: fears and realities. Expert Rev Anti Infect Ther 7: 157-163.
